# Combined Unfolded Principal Component Analysis and Artificial Neural Network for Determination of Ibuprofen in Human Serum by Three-Dimensional Excitation–Emission Matrix Fluorescence Spectroscopy

**Published:** 2018

**Authors:** Gholamreza Bahrami, Hamid Nabiyar, Komail Sadrjavadi, Mohsen Shahlaei

**Affiliations:** a *Medical Biology Research Center, Kermanshah University of Medical Sciences, Kermanshah, Iran. *; b *Student Research Committee, Kermanshah University of Medical Sciences, Kermanshah, Iran. *; c *Pharmaceutical Sciences Research Center*,* School of Pharmacy, Kermanshah University of Medical Sciences, Kermanshah, Iran. *; d *Nano Drug Delivery Research Center*,* School of Pharmacy, Kermanshah University of Medical Sciences, Kermanshah, Iran.*

**Keywords:** Ibuprofen, Excitation-emission fluorescence matrices, Principal component analysis, Artificial neural network, Data Reduction

## Abstract

This study describes a simple and rapid approach of monitoring ibuprofen (IBP). Unfolded principal component analysis-artificial neural network (UPCA-ANN) and excitation-emission spectra resulted from spectrofluorimetry method were combined to develop new model in the determination of IBF in human serum samples. Fluorescence landscapes with excitation wavelengths from 235 to 265 nm and emission wavelengths in the range 300–500 nm were obtained. The figures of merit for the developed model were evaluated. High performance liquid chromatography (HPLC) technique was also used as a standard method. Accuracy of the method was investigated by analysis of the serum samples spiked with various concentration of IBF and an average relative error of prediction of 0.18% was obtained. The results indicated that the proposed method is an interesting alternative to the traditional techniques normally used for determination of IBF such as HPLC.

## Introduction

**Scheme 1:**
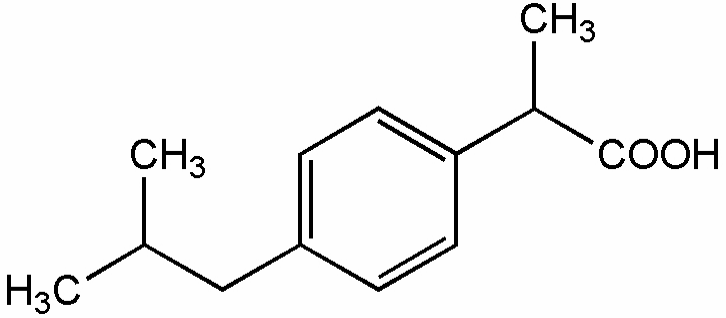
The 2D structure of Ibuprofen

Ibuprofen, [(*R*,*S*)-α-methyl-4- (2-methylpropyl) benzeneacetic acid], (IBP) (Scheme 1) is a non-steroidal anti-inflammatory drug (NSAID) employed in the treatment of pain and inflammation in rheumatic disease and other musculoskeletal disorders (1).

Many methods and techniques have been used for the measurement of IBP, including potentiometric titration (2), flow-injection analysis-FT-IR (3), high-performance liquid chromatography (4, 5), and supercritical fluid chromatography (6).

The role of spectrofluorimetry in the analysis of pharmaceutical compounds has increased. The application of spectrofluorimetry to the analysis of pharmaceutical compounds in biological fluids is advantageous because of the high sensitivity that can be achieved. However, the selectivity is often reduced by extensive spectral overlap or in the presence of matrix interferences. In this context, chemometrics techniques such as principal component analysis (PCA) are useful in circumventing the selectivity problems. PCA is a very useful approach of extracting information from an excitation–emission matrix (EEM), *i.e.* several samples where the fluorescence intensity is depicted as a function of both excitation and emission wavelengths. On the other hand, in recent years, multiway chemometric methods have been introduced for analysis of complex samples. The advantage of using data involving high-dimensional structured information is the higher stability towards interferents and matrix effects, in comparison with first-order methodologies and. Also, in some situations, multiway analysis allows direct separation of the measured signals into the underlying contributions from individual analytes (7). Second order calibration methods is gaining widespread acceptance by the analytical community, as can be detected from literature in relevant analytical, chemometrics, and applied journals. This is due to the variety of second-order instrumental data that are being generated by modern analytical instruments, and to their appeal from the analytical chemistry point of view. Similarly, a multiplicity of mathematical algorithms is available to analytical chemists for the convenient study of this body of information, with applications including environmental, biological, and food analyses (8).

Chemists are often confronted with the problem of extracting information about poorly-known processes from high-dimensional structured data. Discerning the significant patterns in high-dimensional structured data, as a first step to procedure understanding, can be to a great extent facilitated by reducing dimensionality.

The superficial dimensionality of high-dimensional structured data, or the number of individual samples constituting one measurement vector, is often much greater than the intrinsic dimensionality, the number of independent variables underlying the significant nonrandom variations in the samples (9). The problem of dimensionality reduction is strongly connected with feature extraction. Feature extraction refers to recognizing the salient aspects or properties of high-dimensional structured data to facilitate its application in a subsequent task, such as regression or classification (9). Its features are a set of new derived variables, functions of the original independent variables, which efficiently capture the information contained in the original data. 

The most popular technique for feature extraction in chemometrics is principal component analysis (PCA). In practice, principle components (PCs) are often successfully employed as inputs. Even if there is some nonlinearity in original data set, all relevant information are usually contained in the first PCs (10). PCA became efficient algorithm to get rid of possible complications caused by multicollinearity from the independent variables. Reducing the number of inputs to a network reduces the training time and repetition in the input data (11, 12). The use of an artificial neural network (ANN) model with data preprocessing approach, such as PCA and compressing data into scores to quantify complicated and biological mixtures in different analytical samples has been reported (8,10 and 13).

Artificial intelligence consists of different approaches such as ANN and fuzzy logic, applied to solve complicated problems based on human intelligence (14). ANN models represent a technique that deal with uncertainty arising from system complexity and they can be effectively applied to handle uncertainties (15, 16) inherent in absorbance spectra. A typical ANN model is a non-linear computing system consisting of a large number of interconnected processing units (neurons), which simulates human brain learning. 

The outstanding feature of ANN comes from its notable information processing characteristics pertinent primarily to nonlinearity, high parallelism, fault tolerance, as well as learning and generalization capabilities (17, 18). Among different algorithms of ANN, the most generally employed one is the multi layer feed forward neural network. This type of ANN builds a global function approximation and, even if the direct use of a single multi layer feed forward neural network to model a complicated relationship between independent(s) and dependent variables has been proved to be better than conventional techniques, there is a need for further improvement of its performance or generalization capability (19). The performance of a multi layer feed forward neural network depends mostly on data representation (20). One of the main features of data representation is uncorrelation, since correlated data introduce confusion to the neural network during the learning process (21). In addition, many input variables may cause poor generalization performance (22). These difficulties can be handled by combining a feed forward neural network with PCA (23). 

In the present research, the applicability of artificial neural network assisted by principal component analysis was studied. The procedure was based on the recording of excitation-emission fluorescence spectra of IBP in the chloroform and using ANN for its determination in serum. This approach is very precise, sensitive, and applicable to the determination of IBP over wide ranges. Using this method determination can be performed without decreasing the signal-to-noise ratio, and any need to carefully control the experimental conditions. Several synthesis solutions were estimated and the method was validated using biological samples.

## Experimental

*Chemicals and Apparatus*

All reagents and IBF were of analytical grade: chloroform was used as received from Sigma–Aldrich. Stock solutions were prepared by weighing the appropriate amounts of the reagents and dissolving them in chloroform. Working solutions were prepared by diluting stock solutions with chloroform. Serum samples were obtained from fasting and healthy men. It was assumed that the IBF concentration of all these serum samples is zero. Fluorescence spectral measurements were performed on a Perkin-Elmer LS 45 Fluorospectrometer with a 10 mm quartz cuvette at room temperature. The FL WinLab Software (Perkin-Elmer) was applied for measurements spectra recording. 

The instrument consists of two monochromators (excitation and emission), a Xenon light source, a range of ﬁxed width selectable slits, selectable ﬁlters, attenuators and two photomultiplier tubes as detectors. The spectroﬂuorimeter is connected to a PC microcomputer via an IEE serial interface. All measurements were performed in 10 mm quartz cells at room temperature. EEMs were registered in the ranges λ_em_ = 300–500 nm, each 0.5 nm, and λ_ex_ = 235–265 nm, each 1 nm for emission and excitation, respectively. The excitation and emission monochromator slit widths were fixed at 10.0 nm both, and the scanning rate was 600 nm min^−1^.

Stock solutions of the analytes (1 × 10^-3^ M) were prepared by dissolving appropriate amount of IBP in chloroform. Working solutions of lower concentrations were prepared by proper dilution from the stock solution.

*Software*

All calculations were done using MATLAB 7.1 (24). Appropriate m-files for employing unfolded principal component analysis combined with artificial neural network (UPCA-ANN) were written by our group. A useful MATLAB toolbox was developed for easy data manipulation and graphics presentation. This toolbox provides a simple mean of loading the data matrices into the MATLAB working space before running UPCA-ANN. It also allows selecting appropriate recording spectral regions, optimizing the number of factors, calculating the analytical ﬁgures of merit and plotting emission and excitation spectral proﬁles and also pseudo-univariate calibration graphs. This MATLAB toolbox is available from the authors on request. Other calculations were performed using routines developed in our laboratory in the MATLAB environment.

*Procedure*

A 1000 µL mixture of serum and analyte (IBP) was shaked with 2.0 mL of chloroform and 1.0 mL HCl 2M for 5 min and then centrifuged at 7000 rpm for 15 min and procedure was repeated three times. The organic phase was separated and dissolved in 4 mL chloroform and employed for spectroflurometric. The blank solution was prepared using the same procedure as for analytes explained above except that no analyte was added to the serum. All the spectrums were recorded in the excitation range from 235–265 nm (step 2 nm) and in the emission range from 300 to 500 nm (step 0.5 nm). The excitation and emission monochromator slit widths were fixed at 10.0 nm both, and the scanning rate was 600 nm min^−1^.

*Assigning training and test sets*

Three sets of standard solutions (*i.e.* calibration, prediction and validation sets) were prepared. As shown in Table 1, the calibration set contained 40 standard solutions, 9 standard solutions as validation set, and 12 solutions were used in the test set. The respective concentration of IBF in the standard solutions was 0.1 × 10^-7 ^- 47 × 10^-7^.

For preparation of each solution, the required volumes of stock solution were added to a 10.0 mL volumetric flask, and the contents of the flask were diluted to volume with chloroform. 

When a sample produces a J × K data matrix (a second-order tensor), such as an EEM (J = number of emission wavelengths, K = number of excitation wavelengths), the corresponding set obtained by ‘stacking’ the matrices obtained for each of I samples is a three-dimensional or three-way array. Appropriate dimensions of such an array are I × J × K (I = number of samples). The resulted 3D array was unfolded to a two way array. This array was used in order to PCA and then resulted scores splitting to training and test sets. In this step around 20% of the samples (12 out of 61) were selected from unfolded matrix as test set (Table 1) and 15% as validation set (9 out of 61). The best way of assigning test and calibration sets is dividing dataset to guarantee that both sets individually cover the total space occupied by original data set. Ideal splitting of data set is performed in such a way that each of the samples in test set is close to at least one of the samples in the training set. Various methods were used as tools for splitting the whole original data set into the training and test set. According to Tropsha *et al.* the best models would be built when Kennard and Stone algorithm was used (25). For more details see (26, 27). Thus, this algorithm was applied in this study 

(28). 

*Principal Component Analysis (PCA)*

Multivariate calibration methods are important applications in multicomponent spectrophotometry. Let A and C represent the matrices of unfolded fluorescence intensities and the concentration of a set of standard solutions containing serum spiked with IBF, respectively. Then, the resulted matrix has l columns, the following Equation applies:

$A_{m\times 1}=C_{m\times n}K_{n\times 1}$

where K is the coefficient matrix. According to this equation, it is possible to determine the principal components individually with the application of suitable chemometric techniques (29). 

PCR is principal component multivariate mathematical tools, which have been successfully applied to analysis of multicomponent mixtures. As with the more conventional classical least squares method, PCR also need a calibration step where chemometrics model is generated on the basis of the measured spectra and relevant component concentrations of the standard samples. Spectra of the unknown solutions are then compared with the calibration set to predict the concentrations of the validation and subsequently the unknown samples. 

The resulted unfolded matrix was exported to the MATLAB routines for the purpose of PCA. PCA models the maximum directions of variation in a data set by projecting the samples as a swarm of points in a space spanned by PC’s. Each PC is a linear function of a number of original columns of unfolded matrix, resulting in a reduction of the original number of variables. PCs describe, in decreasing order, the most variation among the samples, and because they are calculated to be orthogonal to one another, each PC can be interpreted independently. This allows an overview of the data structure by revealing relationships between the samples as well as the detection of deviating samples. To find these sources of variation, the original data matrix of unfolded EEM, is decomposed into the new spaces such as sample space and the error matrix. The latter represents the variation not explained by the extracted PC’s and is dependent on the problem definition. The approach describing this decomposition is presented as:

A(m,l) = T(m,k)P(k,l)^T ^+ E(m,l)

Where A is the unfolded matrix matrix, T is the scores matrix, P is the loadings matrix, E is the error matrix, m is the number of samples, l is the number of columns in original unfolded matrix, and k is the number of PC’s used. 

In PCR procedure, all calculated scores were collected in a single data matrix and the best subset of PCs was obtained by a stepwise regression.

*Artificial neural network*

**Figure 1:**
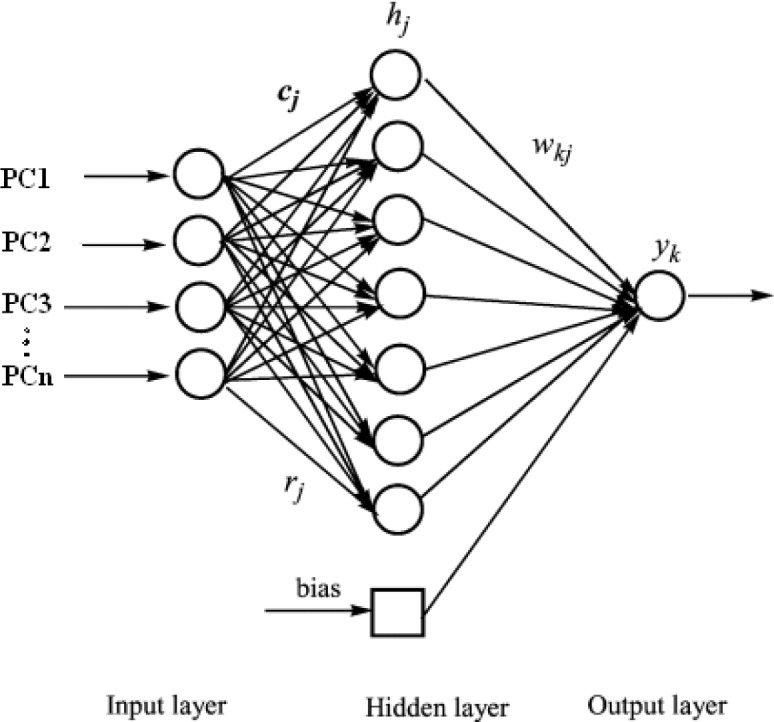
The typical architecture of the ANN

One method to providing a more flexible form of linear regression is to use a feed-forward neural network with error back-propagation learning algorithm. This is a computational system whose design is based on the architecture of biological neural networks and which consists of artificial ‘neurons’ joined so that signals from one neuron can be passed to many others (Figure 1). Clarification of the theory of the artificial neural networks in details has been adequately described elsewhere (30) but little relevant remarks is presented. ANN are parallel computational tools consisting of computing units named neurons and connections between neurons named synapses that are arranged in a series of layers.

Back propagation artificial neural network includes three layers. The first layer namely input layer has n_i_ neurons, and the duty of this layer is reception of information (*i.e.* inputs) and transfers them to all neurons in the next layer called the hidden layer that number of them was indicated by n_h_. The neurons in the hidden layer calculate a weighted sum of the inputs that is subsequently transformed by a linear or non-linear function. The last layer is the output layer and its neurons handle the output from the network and it is the calculated response vector. Duty of synapses is connection of input layer to hidden layer and hidden layer to output layer. The manner in which each node transforms its input depends on the ″weights″ and bias of the node, which are modifiable. On the other hand the output value of each node depends on both the weight, and biases values. In addition, depend on, the weighted sum of all network inputs, which are normally transformed by a nonlinear or linear transform function determining the outputs of the network.

The relation between response, *Y*_o_ of the network and a vector input, *X*_i _can be written as following if number of neurons in the output layer is equal to 1 (same with our condition in here):

$Y_{0}=\sum_{J=1}^{N_{H}}W_{j}f\left(\sum_{I=1}^{N_{I}}W_{\mathrm{jI}}X_{I}+b_{I}\right)$

(1)

Where b_I _is the bias term, W_JI_ is the weight of the connection between the I^th^ neuron of the input layer and the J^th^ neuron of the hidden layer, and f is the transformation function of the hidden layer. In the training process, the weights and bias of the network which are the adjustable parameters of the network are determined from a set of objects, known as training set. 

Through the training of the network, the connection weights are regulated so that error of calculated responses and observed values were minimized. For this, a nonlinear transfer function makes a connection between the inputs and the outputs. Commonly neural network is adjusted, or trained, so that a particular input leads to a speciﬁc target output. There are numerous algorithms available for training ANN models. We used back propagation algorithm here for training of network. In this algorithm several steps for minimizing of networks were performed and the update of weight for the (n + 1) the pattern is given as:

$W_{\mathrm{JI},n+1}=W_{\mathrm{JI},n}+\alpha ∆W_{\mathrm{JI},n}$

 (2)

With using following equation the descent down the error surface is calculated (36):

$∆W_{\mathrm{JI},n}=-\mu \frac{\partial E}{\partial W_{\mathrm{JI},n}}$

 (3)

Where *α* and *μ* are momentum and learning rate, respectively.

With respect to above demonstration, in the ANN some adjustable parameters exist including number of nodes in input and hidden layers, transfer function of hidden and transfer function output layers, momentum, number of iteration for training of network and learning rate that were evaluated by obtaining those which result in minimum in the error of prediction.

As mentioned above in order to avoid overfitting and underfitting, a validation set was used in the ANN modeling. 

All ANN calculations were performed using home-developed scripts using the MATLAB package. 

*Statistical parameters*

For an evaluation of the predictive power of the generated model, the optimized model was applied for the prediction of the IBP values of the test compounds in the test set, which were not used in the calibration procedure. 

For the constructed models, some general statistical parameters were selected to evaluate the prediction ability of the model for IBF concentration. For this case, the predicted IBF concentration of each sample in the prediction step was compared with the experimental IBF concentration (31-33). 

The root mean square error of prediction (RMSEP) is a measurement of the average difference between predicted and experimental values, at the prediction stage (33). The RMSEP can be interpreted as the average prediction error, expressed in the same units as the original response values. The RMSEP Was obtained using the following formula:

$\mathrm{RMSE}={\left[1/n\sum_{i=1}^{n}{\left(\overset{\text{\textasciicircum{}}}{y}-y_{i}\right)}^{2}\right]}^{2}$

 (6)

The second statistical parameter was the relative error of prediction (REP (%)) percent that shows the predictive ability of each component, and is calculated as:

$\mathrm{REP}\left(\%\right)=y/100{\left[1/n\sum_{i=1}^{n}{\left(\overset{\text{\textasciicircum{}}}{y}-y_{i}\right)}^{2}\right]}^{2}$

(7)

where *y*_i_ is the experimental concentration of CLX in the sample i, *y*_i_ represents the predicted CLX concentration in the sample i, y- is the mean of experimental CLX concentration in the prediction set and n is the total number of samples used in the prediction set.

Square of the correlation coefficient (R^2^) is another parameter that was calculated for each model using following formula: 

$R^{2}=\sum_{i=1}^{n}{\left({\overset{\text{\textasciicircum{}}}{y}}_{i}-\bar{y}\right)}^{2}/\sum_{i=1}^{n}\left(y_{i}-{\bar{y}}_{i}\right)$

 (12)

R^2^ is a statistic that will give some information about the goodness of fit of a developed model. Saying another way, the R^2^ is a statistical measure of how well the developed model approximates the real data concentration. An R^2^ of 1 indicates that the regression line perfectly fits the data.

*HPLC procedure*

In order to determine the concentration of IBP in human serum using HPLC, an extraction method was used applying hexane. Serum samples were stored at −40 °C until assay and frozen samples were thawed in water at 37 °C. The serum samples were spiked with appropriate amounts of standard solutions, resulting in an IBP concentration range from 2.43 × 10^-8^ to 2.43 × 10^-8^ M. Aliquots of blank, calibration standard, or test serum samples (100 μL) were pipetted into separate Eppendorf tubes, containing different concentration of IBP. The samples were extracted with 1 mL of hexane, after vortex mixing for 20 s. the organic phase was separated and its evaporation at 40 °C under the nitrogen flow. 

The HPLC system used consisted of two pumps of Shimadzu LC-10A solvent delivery system, a system controller (SCL 10AD), a spectroflurometric detector (RF-551) operated at excitation and emission wavelengths of 267 and 360 nm, respectively. A column oven (CTO-10A), a degasser (DGU-3A) and a data processor (C-R4A) all from Shimadzu, Kyoto, and Japan were applied. The analytical column was a CLC-ODS-3 (MZ, Germany), 125 mm × 4 mm I.D., 5 μm particle size. A mixture of acetonitrile and Triethylamine buffer (47:53) was used as the mobile phase. The column oven temperature was set at 50 °C and the mobile phase was filtered, degassed, and pumped at a flow rate of 1.8 mL/min.

The calibration equation was H = 1.0 × 10 ^-8^c – 0.511 (R^2^ = 0.997), where H is the analyte height and c its concentration in molar. Calibration curves were obtained by linear least-squares regression analysis plotting of peak-height versus the IBP concentrations. For the analysis of real samples containing IBP, appropriate dilutions were made with mobile phase, before filtering and injecting them into the chromatograph.

## Results and Discussion

**Figure 2:**
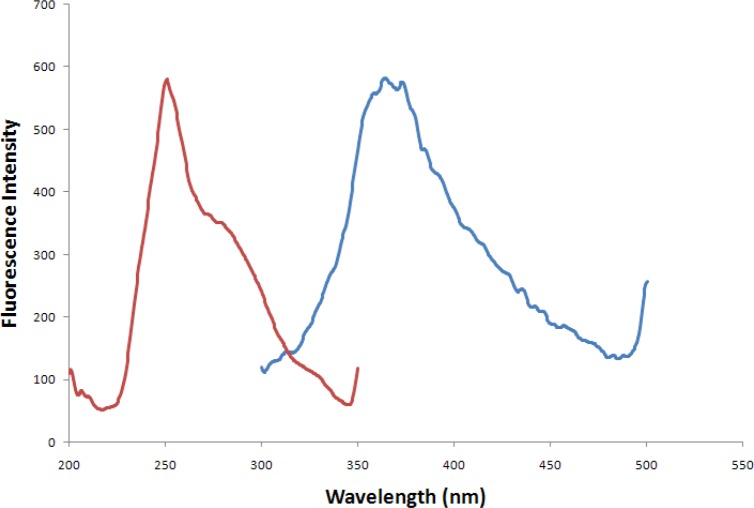
Excitation (Red line) and emission (Blue line) spectra of IBP

IBF exhibits native fluorescence, showing a maximum emission wavelength at 362.5 nm and a maximum excitation wavelength at 251 nm are shown in Figure 2.

**Figure 3:**
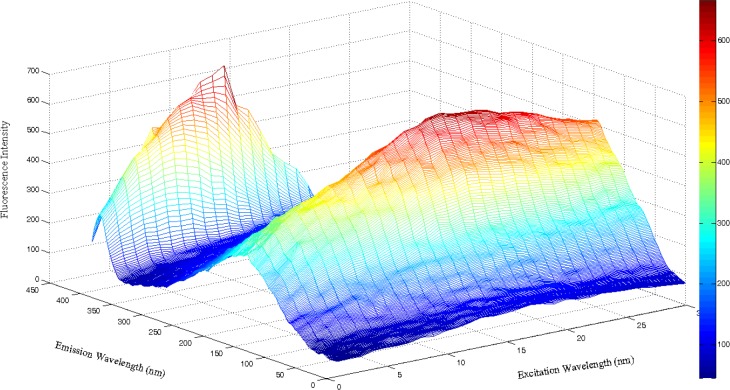
Three-dimensional excitation–emission fluorescence plot for a sample containing 1 × 10 ^-8^ M IBP.

The excitation-emission matrix (EEM) spectra were recorded at excitation wavelengths (λ_ex_) from 235 to 265 nm at regular steps of 1 nm; the emission wavelengths (λ_em_) ranged from 300 to 500 nm at steps of 0.5 nm. Therefore, for each sample, the excitation–emission raw data matrix measured 402 λ_em_ by 30 λ_ex_. As an example, Figure 3 shows a three-dimensional plot of the fluorescence of a typical IBP sample.

**Figure 4:**
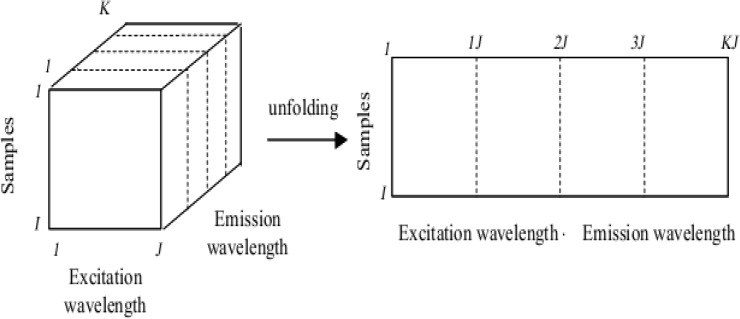
Arrangement of the EEMs in a cube and unfolding by combining the spectral modes.

**Figure 5:**
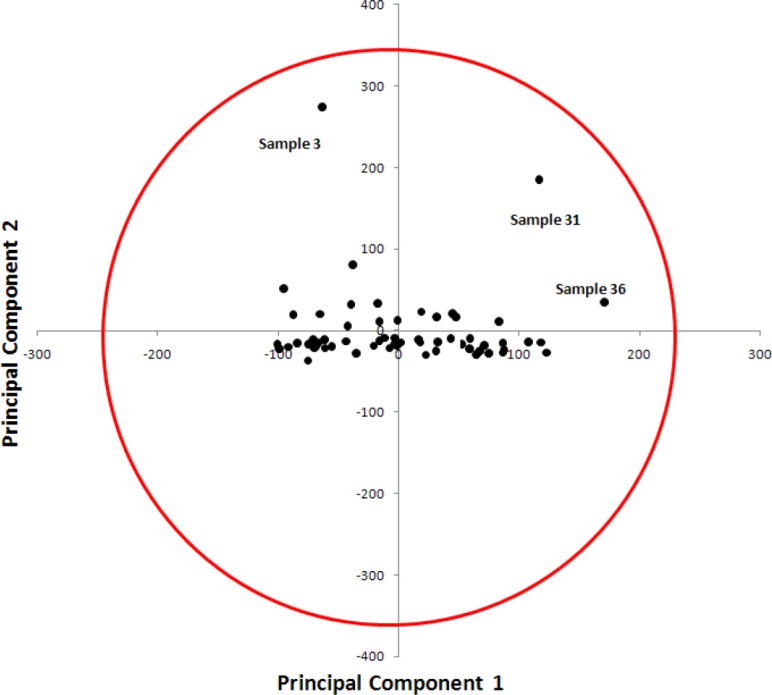
PC_1_–PC_2 _plot for all studied compounds.

**Figure 6:**
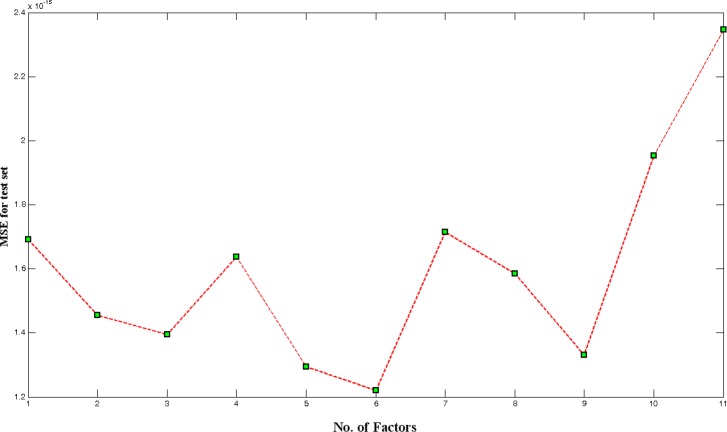
Optimization of number of PCs used in neural network

**Figure 7:**
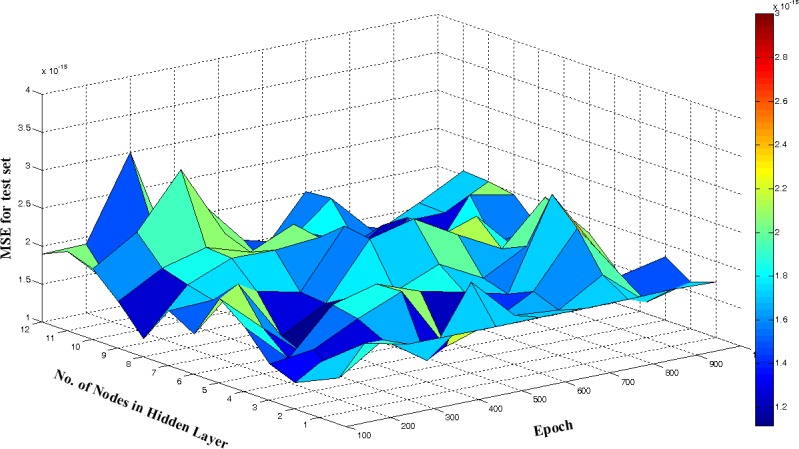
Calculated MSE for test set for the 6 PCs, at different number of hidden nodes and epoch value in ANN model

**Figure 8:**
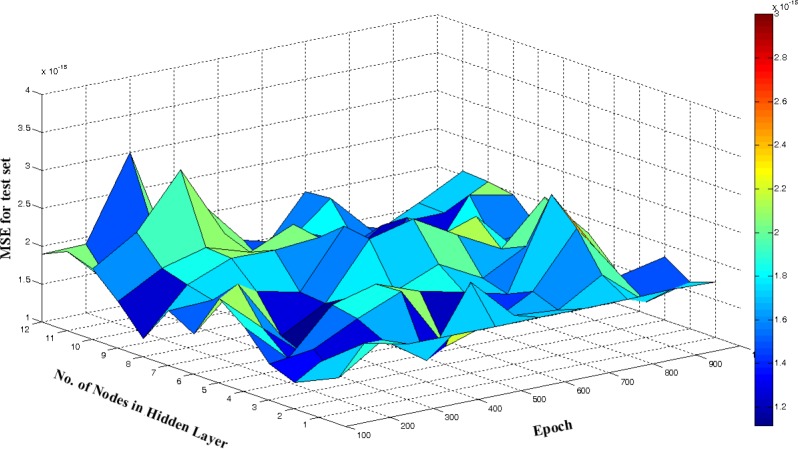
Plot of the concentration of IBP spiked in plasma *vs.* the values predicted by UPCA-ANN for training, validation and test sets

**Figure 9:**
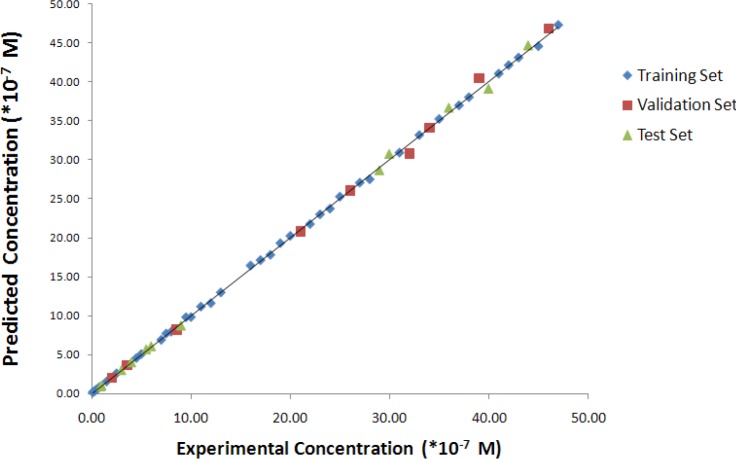
Plot of the predicted residual of IBP by UPCA-ANN procedure for training, validation and test sets

**Figure 10:**
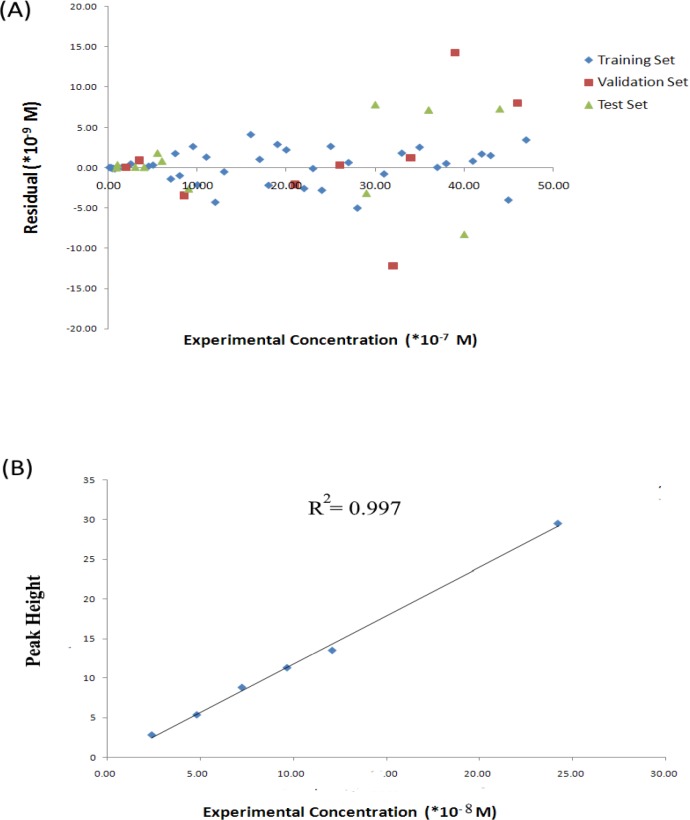
(A) Residuals for all studied sets (B) Calibration curve obtained by HPLC.

**Table 1:** The composition of the calibration and prediction sets solutions for determination of celecoxib in the serum.

**Samples**	**Concentration (** * **10** ^-7^ ** M)**	**Predicted Concentration (** * **10** ^-7^ ** M)**	**REP%**
1	11	11.13	1.20
2	12	11.57	-3.58
3	13	12.95	-0.39
4	1.5	1.51	0.46
5	16	16.41	2.58
6	17	17.10	0.61
7	18	17.78	-1.22
8	19	19.29	1.52
9Ɨ	21	20.79	-1.00
10	22	21.74	-1.17
11	23	22.99	-0.04
12	24	23.72	-1.17
13	25	25.27	1.06
14	2.5	2.55	1.91
15Ɨ	26	26.03	0.12
16	27	27.06	0.24
17	28	27.50	-1.79
18*	29	28.68	-1.09
19	31	30.92	-0.25
20Ɨ	32	30.78	-3.81
21	33	33.18	0.55
22Ɨ	34	34.12	0.35
23	35	35.25	0.73
24Ɨ	3.5	3.59	2.59
25*	36	36.72	1.99
26	37	37.00	0.01
27	38	38.05	0.14
**Samples**	**Concentration (** * **10** ^-7^ ** M)**	**Predicted Concentration (** * **10** ^-7^ ** M)**	**REP%**
28Ɨ	39	40.43	3.66
29	41	41.08	0.20
30	42	42.17	0.41
31	43	43.15	0.35
32*	44	44.73	1.66
33	45	44.60	-0.89
34	4.5	4.52	0.42
35Ɨ	46	46.80	1.74
36	47	47.35	0.74
37*	5.5	5.68	3.31
38	7.5	7.68	2.35
39Ɨ	8.5	8.15	-4.07
40	9.5	9.76	2.77
41	10	9.79	-2.13
42*	1	1.04	3.82
43	0.1	0.10	3.87
44	20	20.22	1.11
45Ɨ	2	2.00	0.13
46	0.2	0.20	0.10
47*	30	30.78	2.61
48*	3	3.01	0.24
49	0.3	0.30	0.59
50*	40	39.17	-2.07
51*	4	4.01	0.14
52	0.4	0.39	-2.58
53	5	5.03	0.66
54	0.5	0.49	-1.34
55*	6	6.08	1.38
**Samples**	**Concentration (** * **10** ^-7^ ** M)**	**Predicted Concentration (** * **10** ^-7^ ** M)**	**REP%**
56	7	6.86	-2.00
57	0.7	0.69	-2.02
58	8	7.90	-1.23
59	0.8	0.80	-0.54
60*	9	8.74	-2.91
61*	0.9	0.90	0.15

* Samples selected as test set.

Ɨ Samples selected as validation set.

**Table 2:** The results of principal component analysis on unfolded excition –emission matrices

**PC No.**	**Eigenvalue**	**Variance Explained**	**Cumulative Variance Captured**
1	4626.63	39.79	39.79
2	2350.70	20.21	60.00
3	957.80	8.24	68.24
4	801.76	6.89	75.13
5	583.61	5.02	80.15
6	500.02	4.30	84.45
7	324.43	2.79	87.24
8	286.83	2.47	89.70
9	221.43	1.90	91.61
10	172.67	1.48	93.09
11	131.93	1.13	94.23
12	87.53	0.75	94.98
13	70.92	0.61	95.59
14	63.22	0.54	96.13
15	46.45	0.40	96.53
16	42.52	0.37	96.90
17	40.21	0.35	97.25
18	28.25	0.24	97.49
19	26.95	0.23	97.72
20	22.54	0.19	97.91

**Table 3:** The statistics for developed neural network model and comparison with HPLC results.

**Parameters**	**Calibration Set**	**Validation Set**	**Prediction Set**
R^2^	0.999	0.998	0.993
R^2^_cv_	0.997		
RMSE (×10^-9 ^M)	2.07	2.07	4.53
RMSE_CV _(×10^-9 ^M)	3.33		
Dynamic Range (×10^-7^ M)	0.1-47		
Limit of Detection (×10^-7^ M)	0.03		
Limit of Quantification (×10^-7^ M)	0.099		

**Table 4:** The predicted concentration of IBP in the studied set and the corresponding percent of recoveries (Rec%) obtained by HPLC^a^^,^

**Sample No.**	**Experimental Concentration**	**Pred. by HPLC**	**REP%**
1	2.43 × 10^-08^	3.29 × 10^-08^	26.16226
2	4.85 × 10^-08^	5.84 × 10^-08^	17.08207
3	7.27 × 10^-08^	9.31 × 10^-08^	21.93105
4	9.69 × 10^-08^	1.18 × 10^-08^	17.90134
5	1.21 × 10^-07^	1.39 × 10^-08^	13.36734
6	2.42 × 10^-08^	3.004 × 10^-08^	19.35251

a The concentrations are in M.

The quality of the multivariate regression techniques is dependent on the different parameters and many factors can change the calibration quality during a multivariate calibration. These factors include (35) (a) non-selectivity problems, (b) the collinearity problem, (c) the optimum number of the calibration samples and the informative spectral regions, and (d) the outlier problem. Proper considering of these factors would be essential to end up with a high calibration power and accurate determination of the IBP in biological samples and to avoid the tedious application of the chemometrics methods.

*Unfold principal component analysis-Artificial neural network (UPCA-ANN)*

As discussed in Method section, a three-dimensional structure (cube) of data was built with the EEMs of the 61 samples. Since the signal had been measured every 0.5 nm, the dimensions of the cube was 61 × 30 × 401 (samples × λ_ex_ × λ_em_). Later, the cube was unfolded by combining the spectral modes (Figure 4). Hence, a matrix of dimensions 61 × 12030 was obtained. Then, PCA was calculated on the unfolded matrix.

All of PCs calculated are reported in Table 2. In this Table, the percent of variances was explained by each PC and the cumulative percent of variances are represented.

The calculated eigenvectors of the covariance matrix are the PC s. These new vectors form a new base which has the following features:

(i) Each PC defines an axis of maximum variance of the original dataset.

(ii) The new axes (PCs directions) are orthogonal, *i.e.*, the information contained in a given PC is uncorrelated to the one contained in the other PCs. In other words, each new axis or PC captures the maximal level of variation in the data not captured by the previous PCs.

(iii) The eigenvalues corresponding to each PC characterize the amount of information (variance) that it explains.

Typically a few PCs, instead of the initial thousands of columns of unfolded cube, will represent the quasi-totality of the excitation-emission intensity information. From the base defined by the PCs, one can estimate the coordinates of the samples in the new representation space. Samples are then normally projected onto the plane PC2/PC1 containing the major part of the original variance. PCA can be used to detect outliers in calibration and validation sets. The scatter plot of PC1 (first principal component or score vector 1) against PC2 (second principal component or score vector 2) reveals evident pattern in the samples studied and facilitate detection of any potential outlier(s). PC1–PC2 plot was depicted in Figure 5 . The following general observations can be made from visual inspection of Figure 5. There is no obvious relationship between PC1 and PC2. The PCA score plot (Figure 5) showed no separation between samples along the first axis (PC1) and PC2. As can be observed, three samples (sample No. 3, 31 and 36) were identified as outliers.

In order to check if these samples could have a strong influence on the PCA, the model was calculated again without outlier samples. The results were almost identical than when the samples were included.

*PC-ANN modeling*

In the next step, a model using a nonlinear regression model, ANN, was built to make a relationship between PCs and concentration of IBP. This model is called UPCA-ANN. Regression method was run on the calibration data, and the concentrations of the analytes in the prediction set were calculated at the optimum number of PCs. 

Overfitting of network takes place when a developed ANN over learns during the training stage of network. An overfitted ANN model may not carry out well on unseen data set due to its lack of generalization capability. In other words, if over-training does happen, contributions of a small subset of the training set solutions may be considered as a major contribution, thus hindering the ability of the developed PC-ANN model to accurately predict the concentration of solution of interest. An efficient way to overcome this problem is the early stopping technique in which the training process is terminated as soon as the overtraining signal appears. This method needs the data set to be divided into three subsets: training set, test set, and validation sets. The training and the validation sets are the norm in the all model training processes. The test set is used to test the trend of the prediction accuracy of the developed ANN model trained at some point of the training stage. At later training stages, the validation error increases. This is the point when the model should cease to be trained to overcome the overfitting problem. To achieve this purpose, the extracted PCs were divided into three sets: training set (65% or 40 samples), validation set (15% or 9 samples), and external prediction (or test) set (20% or 12 samples) (Table 1). Then, the training and validation sets were employed to optimize the network performance. The regression between the UPCA-ANN output and the concentration was estimated for the three sets individually. To build UPCA-ANN models with lower RMSE, the neural code written was run many times, each time run with different number of neurons in hidden layer and/or initial weights. 

In order to select the optimum number of PCs applied in ANN, a cross-validation procedure, leaving out one (LOO) sample at a time, was used. Given the set of 40 calibration samples, the ANN modeling was performed on 40 calibration samples and, using this calibration, the concentration of the IBP in the sample left out during calibration was predicted. This procedure was repeated 40 times until each calibration sample had been left out once. Then, the predicted concentrations were compared with the known concentrations of the reference sample and the root mean square error of LOO-cross validation (RMSEcv) was calculated. The RMSEcv was calculated in the same manner each time a new PC was added to the ANN model. 

One of the most important factors determining quality of generated ANN model is number of PCs selected for model building. If the selected number of PC is lower than optimum number, the derived model is called underfitted model and may not calculate true activity of molecules. On the other hand, if too many PCs are used the network is overﬁtted. Thus, for initial training of network, we chose 5 hidden nodes, learning rate, and momentum equals to 0.5 in 500 epochs. These values were used for finding optimum number of UPCA-ANN components. This optimization was performed by calculation of mean square error for test set (MSEtest). As it is shown in Figure 6, 6PCs were selected as optimum number of PCs.

A response surface methodology was applied to optimize number of neurons in hidden layer and number of epochs. The value of MSEtest was calculated and recorded after every 100 cycles and for a total of 1000 epochs. The calculated values of MSEtest were plotted against the number of nodes in hidden layer and number of epoch, from which the optimum values of these parameters with minimum value of MSE was determined (Figure 7). It can be seen from ﬁgure that 5 neurons in hidden layer and 300 epochs were sufficient for a good performance of the UPCA-ANN.

One of the most important factors for backpropagation learning is the learning rate of the developed ANN as it determines the size of the weight changes. Smaller learning rates slow the learning process, while larger rates lead to the error function to change wildly without continuously improving. Said another way, the learning rate in a parameter that determines the size of the weights adjustment each time the weights are changed during training. Small values for the learning rate cause small weight changes and large values cause large changes.

The best learning rate is not obvious. If the learning rate is 0.0, the ANN will not learn.

To improve the learning procedure a momentum parameter is employed which permits for larger learning rates. The parameter determines how past weight changes affect current weight changes, by making the next weight change in approximately the same direction as the previous one.

A response surface methodology was also applied to optimize learning rate and momentum parameters (30) .The surface plot of MSEtest as a function of linear rate and momentum in five different numbers of nodes in hidden layer is shown in Figure 8. The results show that an ANN with 6 PCs as input variables, 5 nodes in its hidden layer (6-5-1 architecture), learning rate of 0.7, and momentum of 0.2 resulted in the optimum UPCA-ANN performance. The network was trained using calibration samples and it was assessed by prediction set.

The predicted values of concentrations of the studied samples resulted from the optimized PC-RBFNNs procedures are reported in Table 1, in association with relative error of prediction percent (REP%). The plots of predicted concentration versus experimental concentration and the residuals (predicted concentration- experimental concentration) versus experimental concentration value, obtained by the UPCA-ANN modeling, and the random distribution of residuals about zero mean are shown in Figures 9 and 10, respectively. 

Residuals both for all studied sets are distributed normally around zero (the mean value), therefore the nonlinear correlation between concentration and selected PCs is reliable. The plot of calculated versus experimental concentration tells the same theme, adding the information that visually the calculated values appear to capture the experimental values very well.

The developed model was trained using the samples of training and validation sets and it was evaluated by test samples. For a given model, internal validation, although essential and obligatory, does not adequately assure the predictability of a model. In fact, we are strongly persuaded from previous experience that models with high apparent predictability, emphasized only by internal validation approaches, can be unpredictive when confirmed on new compounds not applied in developing the model. Thus, for a stronger assessment of model applicability for prediction on new samples, external validation of the generated model should always be carried out.

*Validation of the method*

In the present study, the quality of the model was assessed by prediction of concentration of IBP in samples of test set. Results are shown in Table 1 and Figures 9 and 10. 

The statistical quantities of the calibration model obtained by UPCA-RBFNN regression, applying on whole spectral range, are reported in Table 3. The good correlation coefficient (0.999 for training set, 0.997 for validation set, and 0.982 for test set) reveals the capability of the model.

The linear dynamic range was 0.1-47 1^-7 ^for IBP. In this report, the detection limit has been estimated from the univariate definition as described by Garcia (7) and Ketterer (36). The EEM for five blank solutions was obtained under condition described. From the UPCA-ANN modeling, the predicted concentrations for IBP were calculated. The standard deviation of the predicted concentrations for IBP was calculated (S_b_). Then, three times the S_b_ for IBP was taken as the detection limit. Detection limit was 0.03 × 10^-7^ M. The number of PCs used to model EEM–concentration is higher than the number of analytes, which can be attributed to the interaction between the serum components. 

The goodness of the fit for the resulted UPCA-ANN model can be measured by cross-validation statistics such as RMSE for cross-validation (RMSEcv), and cross-validated square of correlation coefficient (R^2^_CV_). The root mean square error for training is also included in Table 3 for comparison between two models.

To investigate the prediction ability of the UPCA-RBFNN model, the developed model was used for quantization of the analyte in a separate prediction set solutions that did not have contribution in the model formation step. 

*Precision and accuracy*

The procedures described above were repeated five times within the day to determine the repeatability (intra-day precision) and five times on different days of week to determine the intermediate precision (inter-day precision) of the developed model. The percentage relative standard deviation (RSD%) values were ≤1.91% (intra-day) and ≤1.97% (inter-day) indicating high precision of the methods. Accuracy was evaluated as percentage relative error (*RE*) between the measured mean concentrations and taken concentrations for IBP. Percent relative error or Bias {bias% = [(Concentration found - known concentration) × 100/known concentration]} was calculated at each concentration. The percent relative error values were ≤ 3% (intra-day) and ≤ 1.97% (inter-day) indicating high precision of the methods. Percent relative error (RE%) values of ≤ 2.5% demonstrate the high accuracy of the proposed methods.

*Comparison of the results of developed model with HPLC*

In order to evaluate the results of the developed chemometrics models, a HPLC procedure was also used to analyze IBP in serum solutions. Adequate chromatographic separation was obtained using the system described above. The Serum samples were prepared according to Method section explained above. One component analysis showed that the chromatographic responses were linear in the concentration ranges which were used in calibration step. Retention times were approximately 5.6 min. The set was used to construct the calibration curve for IBP, and the resulted calibration curve was employed to predict the concentration of the IBP in the test set serum solutions. Table 4 and Figure 10 show results of HPLC analysis on the samples.

LOD was approximately 5.19 × 10^-10^ M and LOQ was 1.71 × 10^-9^ M. The standard calibration curves were linear over the concentration ranges of 4.3 × 10^-8 ^to 4.3 × 10^-7^ M. The correlation coefficients for calibration curves were equal to or better than 0.997. 

The predicted concentrations of IBP in studied set by the HPLC method confirm the high prediction ability of the used HPLC method. The reproducibility of the HPLC was nearly the same as that of UPCA-ANN. However, the calculated theoretical LOD of HPLC (5.19 × 10^-10 ^M serum) was lower than those of UPCA-RBFNN (Table 4).

The results of the UPCA-ANN regression method were compared with the results of HPLC as the reference method. The data supports that the prediction ability of the HPLC method is almost the same as that of the UPCA-ANN procedure. However, the differences are not very significant. 

In addition, the Student *t*-test indicated that there is no significant difference between the results of UPCA-ANN and HPLC.

Therefore, it can be concluded that UPCA-ANN model generated give results that are very close to HPLC technique. The measurement simplicity of the UPCA-ANN procedure in combination with its lowest cost is the great advantage of this modeling technique for determination of the IBP in comparison with the chromatographic methods. On the other hand, the more accurate results obtained by HPLC method indicate the advantage of this technique over the developed UPCA-ANN. 

## Conclusion

A UPCA-ANN was employed to analyze the solutions of IBP in the spiked serum using excitation-emission matrix spectra. In order to evaluate the results obtained by this technique, a HPLC method was also applied. An extraction procedure was used to separate the drug from serum and the other interfering components. The accuracy of the developed regression model was validated by spiking standard IBP to serum and recovering the spiked value. It was found that the proposed method, could predict the concentration of IBP with the average percent of relative error equal to 0.18%. By the analysis of drug in serum with five replicate measurements, it was found that UPCA-ANN produced similar accuracy compared to HPLC. Analysis of the IBP in serum by the two methods indicated excellent agreement between the results obtained by both methods. Thus, UPCA-ANN was proposed as a simple, accurate, and more precise method. The results showed that the combination of principal component analysis and artificial neural network is a good tool that can be applied to spectrofluorimetric excitation–emission data to determine IBP in serum samples. 
